# Characterization of commercially available murine fibrosarcoma NCTC-2472 cells both *in vitro* and as a model of bone cancer pain *in vivo*

**DOI:** 10.1371/journal.pone.0309398

**Published:** 2024-08-29

**Authors:** Yuma T. Ortiz, Leila G. Shamir, Lance R. McMahon, Jenny L. Wilkerson

**Affiliations:** 1 Department of Pharmacodynamics, College of Pharmacy, University of Florida, Gainesville, Florida, United States of America; 2 Department of Pharmaceutical Sciences, Jerry H. Hodge School of Pharmacy, Texas Tech University Health Sciences Center, Amarillo, Texas, United States of America; Museu Paraense Emilio Goeldi, BRAZIL

## Abstract

For many cancer patients tumor burden negatively impacts quality of life due to associated pain onset. Neuropathic pain is commonly associated with late cancer stages, and is resultant of tumor metastasis to bone, herein referred to as cancer-induced bone pain. Given the severe impact on quality of life and clinical treatment strategies focusing on symptom management, novel therapeutics are needed to alleviate cancer-induced bone pain and/or reduce cancer burden. In the current study we characterized a commercially available murine fibrosarcoma cell line, NCTC-2472 *in vitro*, which can be used to assess the capacity of novel compounds to impact cellular viability. We found that dimethyl sulfoxide, a known cytotoxic agent and common drug preparation compound, significantly decreased cell viability in a dose-related manner. We then characterized the *in vivo* tumor development and associated pain behavior characteristics following implantation of NCTC-2472 fibrosarcoma into male and female C3H/HeJ mice. The C3H/HeJ strain was utilized as these mice are syngeneic with NCTC-2472 fibrosarcoma and their use reduces potential implantation failure. We found that tumor development in mice resulted in the development of mechanical allodynia but not thermal hyperalgesia. Gabapentin, a clinically relevant analgesic, produced dose-related mechanical allodynia reversal. These studies provide further characterization of a cancer-induced bone pain model that can be used to examine novel compounds as anti-cancer and analgesic therapeutics.

## Introduction

Soft tissue sarcomas include a heterogenous family of tumors with a comparatively low incidence rate internationally, up to 5 cases per 100,000 individuals annually [1]. Despite accounting for < 1% of all cancers, soft tissue sarcomas are highly metastatic, with an estimated 40% - 50% localized tumor (i.e., fibrosarcoma) patients having metastatic growth [2]. Bone metastasis is the third most common metastatic growth site, following the lungs and liver [3, 4]. Fibrosarcoma bone metastasis often results in poor prognosis, with a median overall 6–12 months survival [5, 6]. In addition to poor survivability, severe pain resulting from disease progression and chemotherapeutic treatment seriously impacts patient quality of life [7, 8].

Cancer patients commonly experience neuropathic pain caused by tumor metastasis, known as cancer-induced bone pain (CIBP). As cancer progresses and sarcoma cells come into contact with highly innervated bone tissue [9], tumor invasion into bone occurs, resulting in cytokine, chemokine, and growth factor release. Specifically, this cancerous microenvironment often includes pro-inflammatory mediators such as cyclooxygenase-2 and chemotactic chemokines like C-C motif ligand 2 which are responsible for dendritic cell attraction, as well as cytokine IL-8 which promotes angiogenesis [10]. These ultimately contribute to the activation and damage of nearby afferent nerve fiber processes [4]. This leads to stereotypical changes of the dorsal root ganglia and spinal cord [11] that are associated with central sensitization and result in the altered function of pain-signaling nerve fibers [12]. The result is the development of an often irreversible and long-lasting neuropathic pain that affects up to 66% of late-stage cancer patients [13].

There are numerous preclinical models of tumor burden validated for use *in vivo*, but most have important caveats that must be considered. One of the earliest models of tumor burden utilized immunodeficient mice implanted with human leukemia via tail vein injections. Immunodeficient mice lack functional immune responses, which may alter pain development, maintenance, and analgesic drug assessment [14, 15]. While tumor burden was observed, the wide distribution of metastatic sites in this model is not conducive to the assessment of localized nociceptive behavior [16]. Localized tumor models used to study CIBP commonly utilize direct cell implantation into the marrow space of the femur. This allows for the examination of cancer-induced bone remodeling and changes in primary afferent neurons within a localized region [16, 17]. Despite this advantage, invasive surgery is needed and immunocompromised animals and/or non-syngeneic cell lines are used in some models [18–20]. A CIBP model developed by Wacnik *et al*. utilizes NCTC-2472 sarcoma cells that are implanted into syngeneic C3H/HeJ mouse calcaneus bone, requiring no surgery [17]. It should be noted that NCTC-2472 fibrosarcoma is commonly used in preclinical CIBP models in C3H/HeJ mice. While there are other cell lines syngeneic to the C3H/HeJ mice, such as HCA-1 hepatocarcinoma, the usage of these cell lines are limited to anti-cancer studies and are not used in CIBP models.

The Wacnik et al. CIBP model has been characterized to induce behavioral, morphological, neurochemical, and algogenic sequelae observed in human calcaneus bone cancer [17, 21]. In some studies utilizing this model mice are reported to exhibit both thermal hyperalgesia and mechanical allodynia following tumor implantation, while in other studies only mechanical allodynia is observed [22]. Both mechanical allodynia, or light touch mechanical sensitivity, and thermal hyperalgesia, or increased sensitivity to thermal heat are common pathological pain behavioral symptoms [23]. In this model these pain-related behaviors can be dose-relatedly attenuated via systemic morphine [17]. Importantly, neither *in vitro* NCTC-2472 cell culture properties nor the implanted tumor volume/growth were reported by Wacnik.

Previous studies characterizing the NCTC-2472 cell line focused on isolation [24, 25], clonal analysis [26], transformation [24], and sarcoma-producing capacity [27] rather than on cellular viability. To further characterize this model, we sought to examine both the growth and morphology of commercially available NCTC-2472 cells used in the hind paw CIBP model. In our current study we also sought to determine NCTC-2472 cellular viability *in vitro* in the presence of increasing portions of dimethyl sulfoxide (DMSO), a known cytotoxic agent and a common drug preparation compound. In addition to *in vitro* characterization, in our current study we assessed tumor development following *in vivo* implantation via tumor volume measurements. This characterization may be important in the future assessment of potential anti-tumor drugs. We also sought to examine the presence of mechanical allodynia and thermal hyperalgesia in the *in vivo* CIBP model. Finally, in our current study we assessed the ability of the standard analgesic, gabapentin, to transiently reverse CIBP pain-related behavior. It is the aim of this study to contribute to the standardization of information regarding NCTC-2472 cells and to inform future studies with tumor burden models utilizing these cells.

## Materials and methods

### Cell lines

NCTC-2472 cells were purchased from the American Type Culture Collection (ATCC) (Rockville, MD) and cultured in NCTC-135 medium (Gibco, Waltham, MA) supplemented with 9% (v/v) horse serum (Gibco, Waltham, MA) and 1% (v/v) penicillin-streptomycin antibiotic (Gibco, Waltham, MA). Cells were plated in 100mm x 15mm culture dishes (Fisher Scientific, Pittsburgh, PA). Cells were incubated at 37 °C, 5% CO_2_ in a Heracell 150i CO_2_ incubator (ThermoFisher, Waltham, MA) until 80% confluency. For passaging, cell monolayers were washed once with phosphate buffered saline (PBS) (Gibco, Waltham, MA) and then detached with trypsin (0.25% ethylenediaminetetraacetic acid) (Gibco, Waltham, MA) for 5 min at 37 °C. Cell centrifugation was conducted at 1500 RPM for 5 min utilizing a 5702 R centrifuge (Eppendorf, Enfield, CT).

### Animals

Animal studies were conducted in compliance with the National Institutes of Health Guide for the Care and Use of Laboratory Animals and approved by the Texas Tech University Health Sciences Center Institutional Animal Care and Use Committee. A total of 12 adult male and 12 adult female (21.3–28.2 g upon arrival) C3H/HeJ mice (Jackson Laboratories, Bar Harbor, ME) were used for all *in vivo* experiments. The inbred mouse strain C3H/HeJ is syngeneic to the NCTC-2472 fibrosarcoma cells used in these experiments, meaning that this cell line is derived from this mouse strain and allows these cells to develop tumors with minimal rejection [17, 28]. All mice were housed in a temperature (20–22 °C)-, humidity (55% ± 10%)-, and light-controlled (12-hr light/dark; lights on at 0700) facility approved for use by the Association for Assessment and Accreditation of Laboratory Animal Care. Both food and water were available *ad libitum*. Animals implanted with fibrosarcoma were sacrificed on post-implantation day (PID) 13 as a humane endpoint to minimize the risk of moribundity. Primary euthanasia was done with CO_2_ and with cervical dislocation as the secondary euthanasia method.

### Drugs and dosing

Cell culture grade DMSO utilized *in vitro* was obtained from ATCC (Manassas, VA). Utilizing quarter log dilutions, an exploratory range of DMSO in complete culture media (0.9%– 15.8% DMSO in 200 μL total volume) was used to assess cytotoxic effects. Gabapentin was obtained from Sigma-Aldrich (St. Louis, MO) and was administered intraperitoneally (*ip*). For all treatments, an injection volume of 0.01 ml/g of body mass was used. Gabapentin vehicle was a 1:1:18 ratio of tween-80, propylene glycol, and saline, respectively. Cumulative doses of gabapentin (10–100 mg/kg) or vehicle were given a 60 min absorption period prior to von Frey tests. This dose range was based upon previous work [29]. A 72 hr minimum washout period was imposed between testing days as described previously [30, 31]. Treatments were carried out via cumulative dosing on two separate days at PID 3 and PID 7. Cumulative dosing was determined optimal for these studies to assess gabapentin-induced analgesia following tumor implantation into the calcaneus bone, while minimizing the impact of rapid tumor growth observed from PID 9 through PID 13. In addition, cumulative dosing decreased the overall number of animals used in these experiments.

### Cellular proliferation, viability, and morphology

The proliferative viability of cells was assessed via measurements of cellular density both at plate seeding and at passaging with a standardized plating density of 3 × 10^4^ cells/mL. Density measurements were used to calculate cell doubling times and to assess any changes to growth efficiency over time by calculating the cumulative population doubling level (CPDL). The CPDL measurements are calculated using the formula CPDL = ln (N_f_/N_i_) ln_2_, where N_i_ is the initial seeding cell density, N_f_ is the final density at passaging, and ln is the natural log [32]. Cumulative levels were obtained by adding the calculated doubling level of the current passage with the doubling levels of the previous passages.

Cellular viability in the presence of the cytotoxic agent DMSO was assessed utilizing 3-(4,5-dimethylthiazol-2-yl)-5-(3-carboxymethoxyphenyl)-2-(4-sulfophenyl)-2H-tetra-zolium (MTS) colorimetric plate readings. The MTS assay is a colorimetric assay of cellular viability that uses MTS (Abcam, Cambridge, UK) as a reagent that is reduced by viable mammalian cells to generate a colored formazan dye that is soluble in culture media. Following kit protocols, cells were seeded into 96-well plates (Corning Incorporated, Kennebunk, ME) at a density of 62.5 cells/μL and were incubated overnight (approximately 16 hrs) to ensure adherence of cells to plates. Cells were then treated with either vehicle (complete media) or DMSO at increasing percentages and incubated for 72 hrs at 37 °C with a 5% CO_2_ atmosphere. Following this incubation period, 20 μL of MTS reagent was added to each control and treatment well. The 96-well plates were then incubated for 4 hrs at 37 °C with a 5% CO_2_ atmosphere. Absorbency measurements were taken after the 4 hr incubation period by a microplate spectrophotometer (BioTek, Winooski, VT) at 490 nm. Each assay was performed in 6 replicate wells for each condition. The addition of cells, treatment, and MTS reagent in 96-well plates were done using a 12-channel multi-channel pipette (ThermoScientific, Waltham, MA) to minimize pipetting error.

Observations and images were captured utilizing an EVOS inverted imaging microscope (Invitrogen, Waltham, MA). All cell images are taken at either 10x or 40x magnification with included scale bars indicating 400 μm or 100 μm respectively. Observations on the effects of over confluency utilized plates that were allowed to exceed the maintained confluency limit of 80%. These plates were allowed to reach a minimum of 100% density with only fresh media being supplemented.

### Fibrosarcoma cell preparation and implantation

Immediately prior to implantation, fibrosarcoma cells were prepared as previously described [17]. In brief, NCTC-2472 cells were trypsinized, pelleted, resuspended, pelleted a second time, and then resuspended in fresh PBS for implantation. Centrifugation of cells was conducted at 1,500 RPM for 5 min.

Mice were placed within an enclosed anesthesia chamber with isoflurane (induction at 5% vol. followed by 2% vol. in oxygen for sustained anesthesia) in preparation for cell implantation. Once the mice demonstrated non-responsiveness to paw pinch and a surgical plane of anesthesia was confirmed, mice were removed from the chamber and fitted with a nose cone to continue anesthetic delivery for the duration of the implantation. The implantation procedure utilized for this study is previously described [17]. Fibrosarcoma cells (2 × 10^5^ cells/μL) in a 10 uL volume of PBS were injected unilaterally into the right heel using a 29-gauge single-use needle attached to a 25 μL glass syringe (Hamilton Company, Reno, NV) to manually bore into the calcaneus bone. Sham mice underwent an identical procedure with the exception that PBS alone was injected.

### Tumor development and assessment of nociception

#### Tumor volume

NCTC-2472 fibrosarcoma cells were localized to the calcaneus area as indicated by formation of a mass in the region of the injection site. Due to the spheroidal shape of the fibrosarcoma tumor, relative tumor volumes were calculated using length and width measurements taken via digital calipers. These measurements were then used to calculate the volume of an ellipsoid using the formula as described in the xenograft tumor model [33] where V = w^2^ × l/2, where V is tumor volume, w is width, and l is length. Measurements were taken daily throughout the course of the *in vivo* experiments to track changes in volume.

#### Mechanical allodynia

Before sarcoma or PBS implantation, mice were habituated to the von Frey testing environment for four consecutive days in 30 min sessions prior to the first testing session. Pre-implantation baselines were measured as previously described using von Frey monofilaments (North Coast Medical, Morgan Hills, CA) to establish responses to light mechanical touch before sarcoma implantation or PBS injection [23, 34]. During von Frey testing, mice were placed on top of a wire mesh screen, with spaces 0.5 mm apart. Mice were unrestrained and were singly placed under an inverted wire mesh cup (8 cm diameter, 15 cm height) and allowed to acclimate to the apparatus for 30 min before testing. The von Frey test utilizes a series of calibrated monofilaments, (0.4–4.0 g stimulus intensity) applied to the left and right plantar surface of the hind paws utilizing the “up-down” method [35]. Monofilament application was done in a manner avoiding sequential application to the same paw (i.e., left, right, left, right as opposed to left, left, left, right). Reactive licking, lifting, clutching, or flicking of the paw upon filament application was considered a response. Five responses out of five monofilament stimulations were coded as the minimum force required to elicit responses within the von Frey assay. Reported measurements from the von Frey assay are mean response thresholds collected from the right hind paw of each subject as fibrosarcoma cell implantation produced ipsilateral allodynia, with no presentation of mechanical allodynia within the contralateral paw. Mechanical allodynia assessments were conducted by observers blinded to treatment conditions.

#### Thermal hyperalgesia

Mice were assessed for acute nociception of thermal stimuli via the hot plate latency assay as previously described [30]. Mice were placed on a heated (52 °C) enclosed Hot Plate Analgesia Meter (Columbus Instruments, Columbus, OH). The latency to jump, lick, lift, clutch, or flick a hind paw was recorded. A 30 sec cut-off was used to prevent tissue damage [31]. Hot plate latencies were assessed a minimum of 1 hr following von Frey testing to avoid confounding measurements. Thermal hyperalgesia assessments were conducted by observers blinded to treatment conditions.

### Data analysis

Data are presented as mean ± standard error of the mean (SEM) of 6 replicates for *in vitro* experiments and 8 replicates for *in vivo* experiments unless stated otherwise. For quantitative analysis of the differences among the mean values between groups, data were analyzed using a repeated measures one-way analysis of variance (ANOVA) with Dunett’s post hoc multiple comparison test through Graphpad Prism software (v10.1.1). A value of *p* < 0.05 was considered statistically significant. As no significant sex effect was observed among behavioral assays, data obtained from males and females were collapsed. If the mean effect of a treatment did not produce a 50% or greater effect *in vivo*, an effective dose (ED)_50_ value was not generated. If the mean effect of treatment did not produce a 50% or greater effect *in vitro*, an inhibitory concentration (IC)_50_ value was not generated. When the mean effect of a drug in an experiment was greater than 50%, the respective ED_50_ or IC_50_ values and corresponding 95% confidence limits were calculated using linear regression, where slopes were allowed to vary [36].

## Results

### Cell morphology and apoptosis

**Fig 1:**
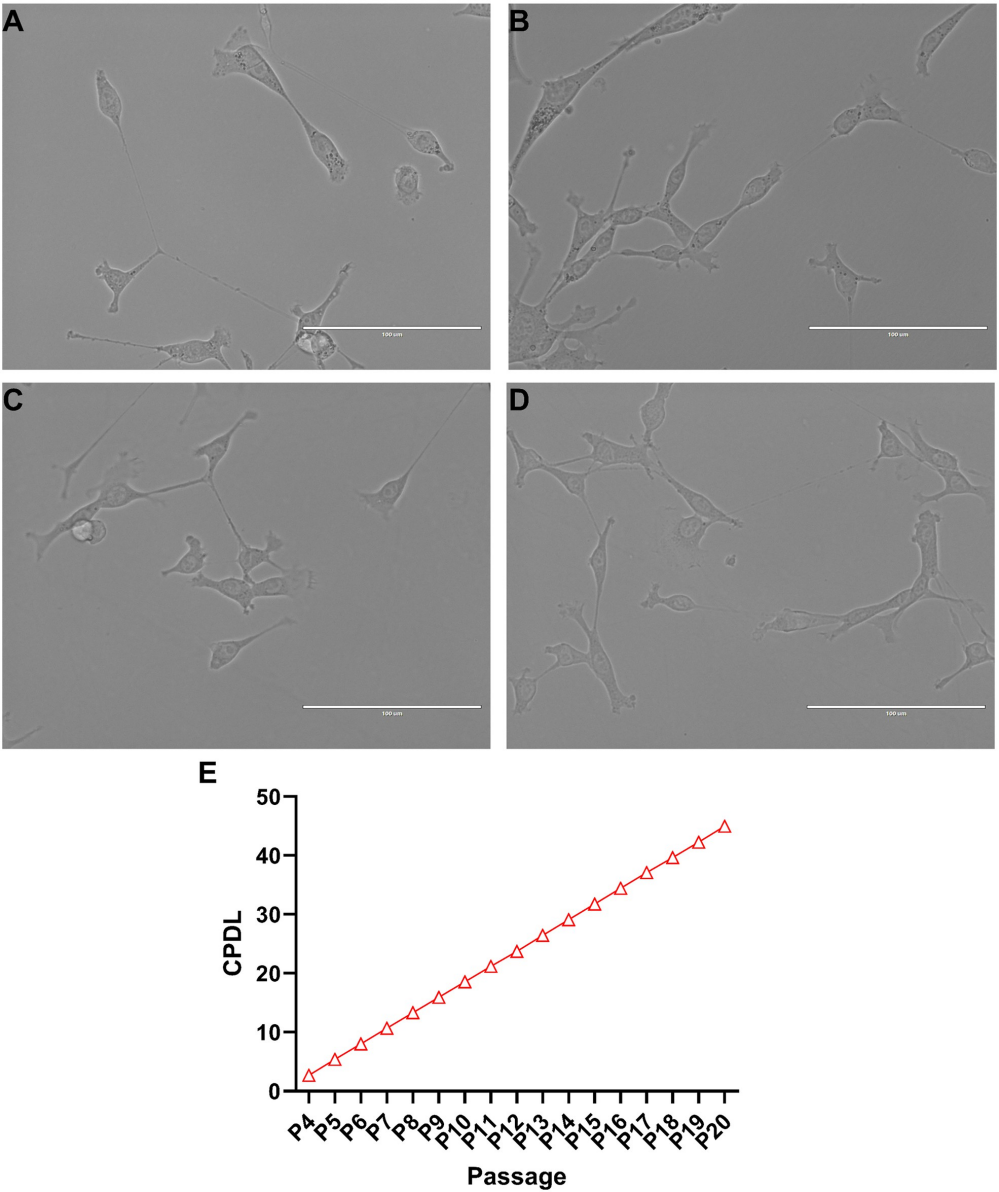
Culture of NCTC-2472 fibrosarcoma cells and identification of cumulative population doubling level (CPDL). Stereotypical fibroblast morphology is exhibited among NCTC-2472 fibrosarcoma (a) with a spindle shape and bipolar presentation. Light microscopy images collected across early passages (passage 5, b), moderate passages (passage 10, c), and late passages (passage 20, d). Fibroblasts exhibited little to no change in morphology despite prolonged culture. Images were taken at 40x magnification at 50% dish confluency. Scale bar represents 100 μM. Cumulative growth curve of NCTC-2472 fibrosarcoma (e) as calculated from passage 4 to passage 20. Abscissae: passage number; ordinates: calculated CPDL value. Data reflect mean ± SEM, n = 6.

NCTC-2472 fibrosarcoma cells did not display prominent morphological markers indicative of apoptosis in regular culture. Throughout continued cell passaging, NCTC-2472 cells exhibited typical cellular morphology associated with fibroblast type cells that include branched cytoplasmic extensions and a general spindle shape body pattern with bipolar presentation (Fig 1a). There was no observed shrinking of the nucleus, no cellular blebbing, minimal presence of cellular debris, and no loss of adherence in low passage numbers (Fig 1b), moderate passage numbers (Fig 1c), or in high passage numbers (Fig 1d) amongst any cells.

To determine the self-renewal capacity of NCTC-2472 fibroblasts, we measured and calculated the proliferative ability of the cells via CPDL measurements (Fig 1e). A consistently linear increase in the rate of cell proliferation was observed and indicated that despite prolonged passaging, there was no impact on cellular proliferation.

**Fig 2:**
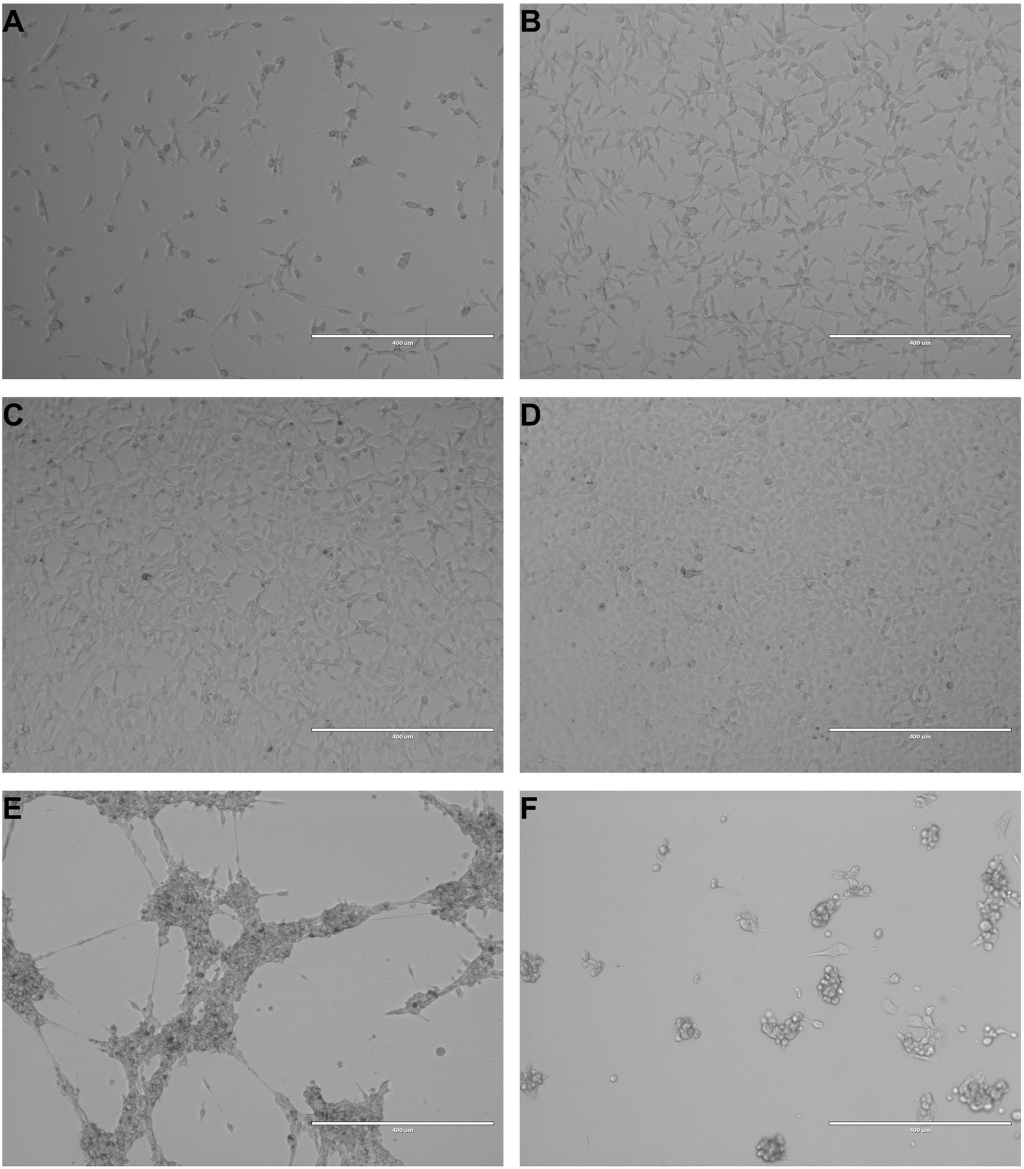
Images of NCTC-2472 cells at various levels of confluency and in poor health. Images are representative of NCTC-2472 fibrosarcoma cells at increasing levels of dish confluency at 20% confluency (a), 50% confluency (b), 80% confluency (c), and 100% confluency (d). Upon surpassing 100% confluency, cells began to clump together into large, multi-cellular strands (e). While NCTC-2472 are robust, lack of fresh nutrients will induce a poor health state (f) with observed cellular shrinkage, blebbing, and loss of adherence. All images were taken at 10x magnification. Scale bar represents 400 μM.

Dish confluency limits were maintained at 80%, with cells passaged upon reaching the imposed confluency limit. Dishes used to assess the effects of over confluency were allowed to reach and exceed 100% confluency. Fig 2 depicts plates at various confluency levels from 20% (Fig 2a), 50% (Fig 2b), 80% (Fig 2c), and approximately 100% (Fig 2d). NCTC-2472 cells that were allowed to surpass 100% confluency initially exhibited minimal contact growth inhibition as cells were observed to layer on top of the original 2-dimensional monolayer, though shortly thereafter, cells were observed to mass together (Fig 2e).

While NCTC-2472 cells generally were not negatively affected by prolonged passaging or over confluency, a limited number of cells did exhibit poor health or death as indicated by hallmark changes in morphology. Primarily the result of unrefreshed media, NCTC-2472 cells in poor health exhibited shrinkage, blebbing, loss of adherence, and the presence of fragmented cellular debris (Fig 2f).

### Cytotoxic effects of DMSO

**Fig 3:**
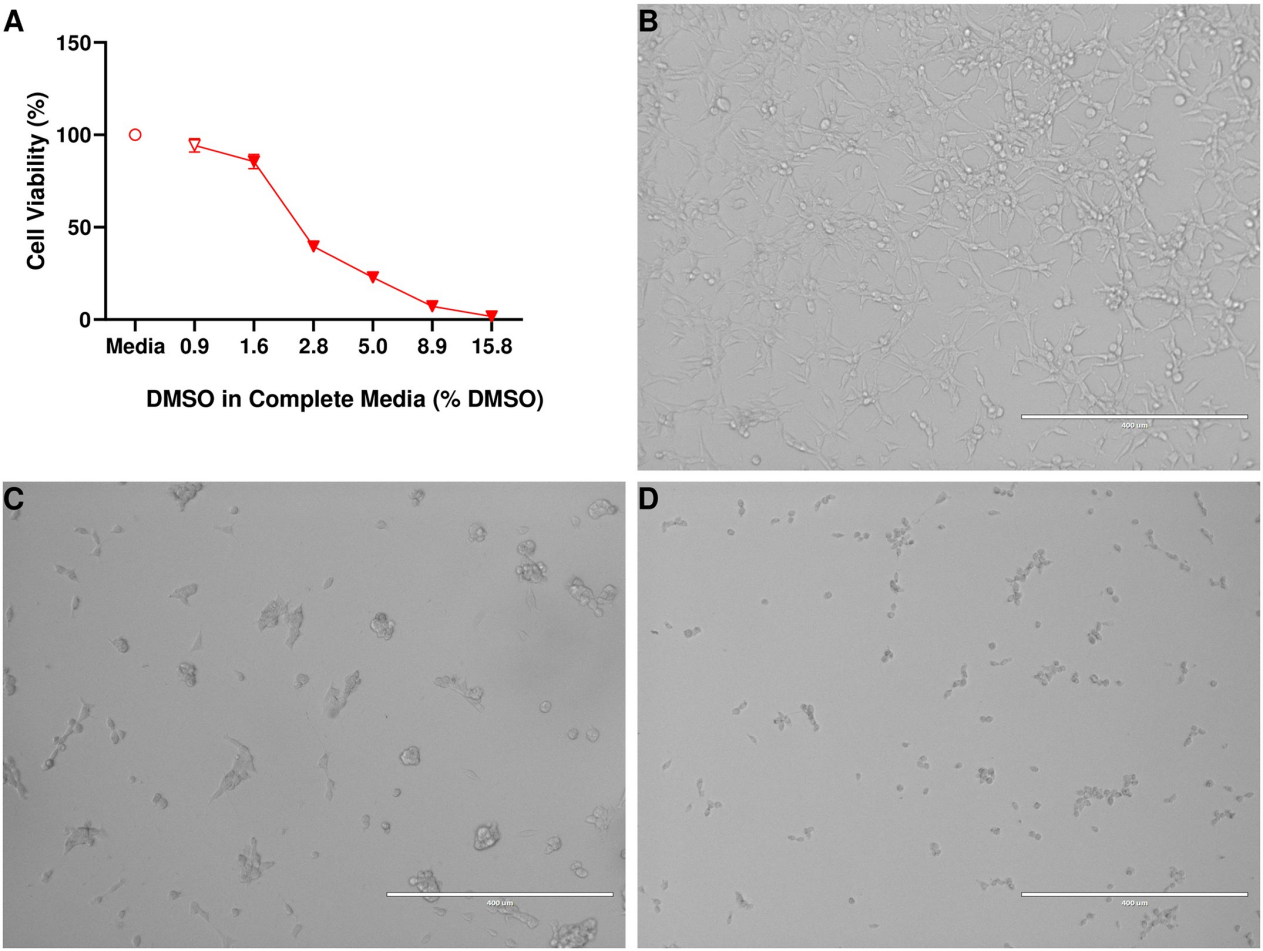
Impact of DMSO on cellular viability of NCTC-2472 fibrosarcoma and representative images. DMSO was found to impact cellular viability of NCTC-2472 fibrosarcoma (a) in a dose-related manner. Images are representative of fibrosarcoma morphology at increasing levels of DMSO present at 0.9% DMSO (b), 2.8% DMSO (c), and 15.8% DMSO (d). Cells in poor health exhibited cellular shrinkage, blebbing, and loss of adherence. All images were taken at 10x magnification. Scale bar represents 400 μM. Abscissae: % DMSO present in 200 μL volume; ordinates: cellular viability of NCTC-2472 fibrosarcoma as a percentage of vehicle (media) treatment. Filled data points indicate a significant difference from vehicle treatment (*p* < 0.01). Data reflect mean ± SEM, n = 6.

Administration of DMSO (Fig 3a) was found to significantly reduce the viability of NCTC-2472 fibrosarcoma (*F* (1.43, 7.17) = 494, *p* < 0.0001) with a calculated IC_50_ value of IC_50_ = 2.93% (2.63%– 3.26%). Representative images of cellular morphology at 0.9% (Fig 3b), 2.8% (Fig 3c), and 15.8% (Fig 3d) DMSO in media show the cytotoxic effects of DMSO in NCTC-2472 fibrosarcoma. Fibrosarcoma treated with DMSO exhibited morphological characteristics such as shrinkage, blebbing, loss of adherence, and the presence of fragmented cellular debris.

### Calcaneus implantation of fibrosarcoma cells, tumor formation, and attenuation of pain behavior

**Fig 4:**
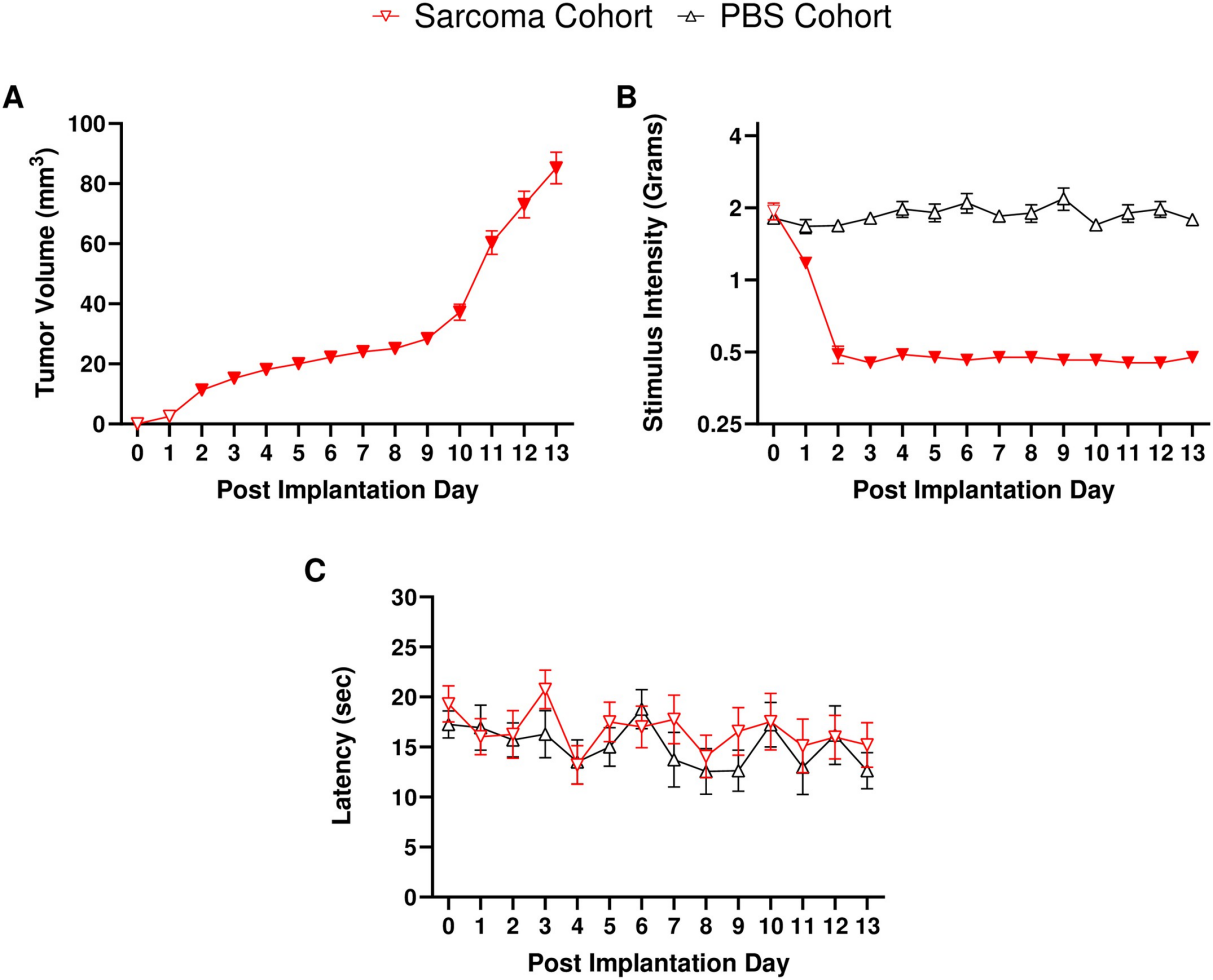
NCTC-2472 fibrosarcoma implantation results in tumor development and mechanical allodynia, though no thermal hyperalgesia is observed. Abscissae: days post implantation; ordinates: (a) Calculated tumor volumes as measured daily, (b) minimum force threshold in grams required to elicit a paw withdrawal response, (c) latency to respond in the hot plate assay. Filled data points indicate a significant difference from PID 0 baseline measurements (*p* < 0.05). Data reflect mean ± SEM, n = 16 (a, b), n = 8 (c).

As this model of hind paw CIBP has not been utilized in our laboratory previously, we sought to characterize the observed behavioral aspects of this pathology. Before sarcoma implantation or PBS injections, mice displayed similar baseline responses in the von Frey assay (*p* = 0.873) and the hot plate latency assay (*p* = 0.46). Following either the implantation of NCTC-2472 fibrosarcoma cells or the injection of PBS into the calcaneus bone of mice, daily measurements of tumor volume (Fig 4a), mechanical allodynia (Fig 4b), and thermal hyperalgesia (Fig 4c) were recorded through PID 13. Tumor volume measurements (Fig 4a) showed significant (*F* (1.57, 17.31) = 74.81, *p* < 0.0001) and slow growth between PID 3 and PID 8, with a period of rapid growth from PID 9 to PID13. Tumor volumes were not recorded for PBS injected mice as they did not exhibit any tumor formation.

Mechanical allodynia (Fig 4b) was assessed via the von Frey assay following NCTC-2472 implantation. A clear and significant onset of tumor-induced mechanical allodynia (*F* (2.97, 68.23) = 107.6, *p* < 0.0001) was observed in sarcoma implanted mice on PID 2 –PID 13, as compared to pre-implantation baselines. PBS injected controls did not display significantly different response thresholds from pre-implantation baselines (*p* = 0.202) across the 13-day time course, indicative that unilateral PBS injection did not produce mechanical allodynia.

Thermal hyperalgesia was assessed via the hot plate latency assay (Fig 4c). However, there was no significant difference observed among fibrosarcoma and PBS animals (*p* = 0.327, *p* = 0.398, respectively) compared to baseline measures, indicating a lack of thermal hyperalgesia development in either cohort.

**Fig 5:**
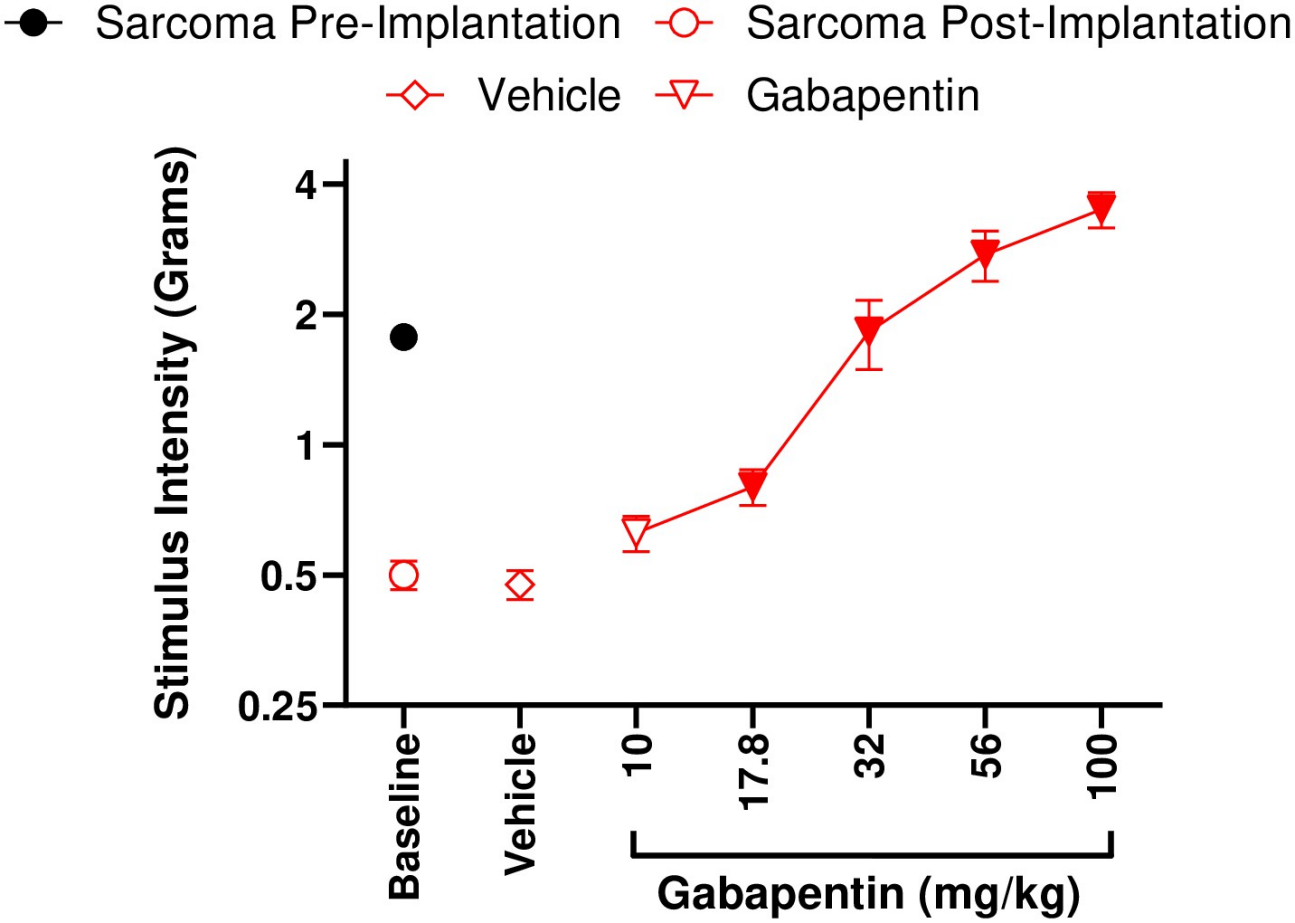
Mechanical allodynia that develops as a result of NCTC-2472 fibrosarcoma implantation can be dose-relatedly attenuated with gabapentin. Abscissae: dose of gabapentin; ordinates: minimum force threshold in grams required to elicit a paw withdrawal response. Filled data points indicate a significant difference from post-implantation baselines (*p* < 0.05). Data reflect mean ± SEM, n = 8.

Following our observations of robust mechanical allodynia induced by tumor development, we sought to pharmacologically attenuate mechanical allodynia. Repeated *ip* vehicle administration, as utilized in our cumulative dosing procedure, did not impart any effect on mechanical allodynia (S1 Fig) in either the PBS (*p* = 0.418) or sarcoma (*p* = 0.827) cohorts, respectively. Gabapentin is used clinically as a neuropathic pain therapeutic. Gabapentin (17.8–100 mg/kg, *ip*) dose-relatedly (Fig 5) attenuated mechanical allodynia (*F* (2.49, 17.45) = 32.99, *p* < 0.0001) in mice implanted with fibrosarcoma in a dose-related manner compared to vehicle von Frey measurements. Vehicle did not attenuate mechanical allodynia as compared to post-implantation von Frey measurements (*p* = 0.685). The calculated ED_50_ of gabapentin to attenuate mechanical allodynia in this model was ED_50_ = 32.41 mg/kg (28.64–36.68). Gabapentin was also found to significantly increase (*F* (1.851, 12.96) = 7.919, *p* = 0.02) the response thresholds of PBS mice at a 100 mg/kg dose (S2 Fig).

## Discussion

In the present study calcaneus NCTC-2472 murine fibrosarcoma cell implantation into syngeneic C3H/HeJ produced mechanical allodynia associated with measurable local tumor development starting at PID 2, lasting to our imposed PID 13 humane endpoint. Although the development of thermal hyperalgesia has been observed in a few previous studies utilizing this hind paw CIBP model we do not see any evidence of these behaviors in our current study [17, 22, 37–39]. However, we observed tumor development and mechanical allodynia onset in a similar manner as reported by Wacnik and colleagues (i.e., approximate onset at PID 3) [17]. We also found that gabapentin dose-relatedly attenuated fibrosarcoma-induced mechanical allodynia.

Although here we did not observe any form of evoked pain behavior other than mechanical allodynia, in some studies, thermal hyperalgesia has been found to follow fibrosarcoma implantation [17, 22, 37–39]. In some of these studies surgery was used to expose the injection site [22]. The current intra-calcaneus model of CIBP improves upon the surgical implantation method by allowing local osteolytic tumor growth within the calcaneus bone. Importantly, regardless of injection method or measures of evoked pain, mechanical allodynia is consistently present in all of these fibrosarcoma bone pain studies [17, 22, 37–39].

One possible reason behind the different evoked pain manifestations may be due to of varying interactions between afferent nerve fibers and local tumor presence, as an invasion into the bone by fibrosarcoma injures the processes of these afferent nerve fibers and leads to changes of the dorsal root ganglia and spinal cord associated with central sensitization [11, 12]. The onset of mechanical allodynia following implantation results from peripheral nerve hyperexcitability and enhanced synaptic efficacy at low-intensity mechanosensitive Aβ fibers [12]. However, thermal hyperalgesia involves nociceptive C fibers, which may also experience enhanced synaptic efficacy and alteration in function under neuropathic pain conditions [12, 40]. It should be noted that the C3H/HeJ strain has a mutation at Toll Like Receptor 4 that leaves the mice susceptible to infection from gram-negative bacteria, though this would have minimal impact as cancer effects on health differ to those due to bacterial infection. Additionally, despite this deficiency, C3H/HeJ mice still exhibit behavioral pain states in inflammatory, cancer, and neuropathic pain models [41]. Experimenter differences, such as hot plate temperature, amount of time allowed on the hot plate, stress level of the animal, and the ambient room temperature may contribute to the observed differences in evoked pain behavior. Although not studied here, the use of non-evoked pain depressed behavior may also be a useful measure to examine fibrosarcoma bone pain [29, 42].

Here we characterized the morphology and growth characteristics of the NCTC-2472 cell line via observations of cell health across various passages and confluency levels. Calculated CPDL paired with the apparent robustness of NCTC-2472 fibrosarcoma indicated that cellular viability was consistently high regardless of prolonged repeated passaging and high confluency levels. Despite this, it is recommended that confluency limits be set for the fibrosarcoma cell line to ensure consistency in passaging. Additionally, while the commercially available NCTC-2472 fibrosarcoma cell line was used for this study, a potential limitation is the lack of primary fibrosarcoma cells being included in these studies to provide representation of primary tumors *in vivo* and *in vitro*.

To assess the effects of cytotoxic compounds, DMSO was administered to cultures of NCTC-2472 fibrosarcoma and the resultant impacts on cellular viability were assessed with the MTS assay. It was observed that increasing amounts of DMSO resulted in increased severity of cytotoxic effects. Representative images show that at sufficient amounts, DMSO treatment can result in morphological changes that are associated with poor cell health that include loss of adherence, shrinkage, and cellular blebbing. These findings establish poor health characteristics of NCTC-2472 cells that would likely be expected in the presence of cytotoxic compounds. Additionally, this provides valuable insight into future studies that may utilize DMSO in preparation of compounds for testing with NCTC-2472 fibrosarcoma *in vitro*.

While the NCTC-2472 cell line has been used regularly in studies assessing tumor burden and cancer-associated pain [22], literature has not organized essential characteristics of these fibrosarcoma cells *in vitro*. Those that do have focused primarily on isolation [24, 25], clonal analysis [26], transformation [24], and sarcoma-producing capacity [27], all of which have been published either when the cell line was first isolated and transformed, or shortly thereafter [43]. The lack of such organized information presents challenges to those looking to utilize NCTC-2472 cells for both *in vitro* and *in vivo* fibrosarcoma studies. The present study shows that NCTC-2472 fibrosarcoma cells expectedly exhibit stereotypical characteristics associated with cancerous cell lines, including quick doubling times, robust proliferative efficiency, lack of contact inhibition, and no apparent replicative exhaustion. Similar to prior *in vitro* studies, previous *in vivo* studies utilizing NCTC-2472 cells lacked some useful information, namely tumor burden growth patterns. This current study expands upon previous NCTC-2472 studies with additional characterization of *in vitro* cell viability and morphology as well as *in vivo* tumor burden development. The NCTC-2472 fibrosarcoma line is a well-established cell line that has continuously been used in models of CIBP. However, NCTC-2472 fibrosarcoma are rarely used to assess novel compound anti-cancer activity *in vitro* via cellular viability assessments such as the MTS assay. The current study supports the use of the NCTC-2472 fibrosarcoma cell line in vitro and in C3H/HeJ mice in vivo as a promising testbed to screen compounds that may have dual anti-cancer and analgesic properties.

## Supporting information

S1 Fig Repeated *ip* administration does not impact mechanical allodynia. Mechanical allodynia is not impacted by the act of repeated *ip* injections in repeated administration experiments and any effects on mechanical allodynia are solely an effect of the compound used for treatment. Abscissae: vehicle dose; ordinate: minimum force threshold in grams required to elicit a paw withdrawal response. Data reflect mean ± SEM, n = 8.
(TIF)

S2 Fig Gabapentin in the cancer-induced bone pain model. Gabapentin was found to significantly produce analgesic effects at 100 mg/kg Gabapentin via cumulative dosing in the PBS implanted cohort. Abscissae: vehicle dose; ordinate: minimum force threshold in grams required to elicit a paw withdrawal response. Filled data points indicate a significant difference from vehicle treatment (p < 0.05). Data reflect mean ± SEM, n = 8.
(TIF)
