# Supplementary figures and images for: Characterization of commercially available murine fibrosarcoma NCTC-2472 cells both in vitro and as a model of bone cancer pain in vivo
Source: PLoS One. 2024 Aug 29;19(8):e0309398. doi: 10.1371/journal.pone.0309398 (PMC11361427; doi:10.1371/journal.pone.0309398)

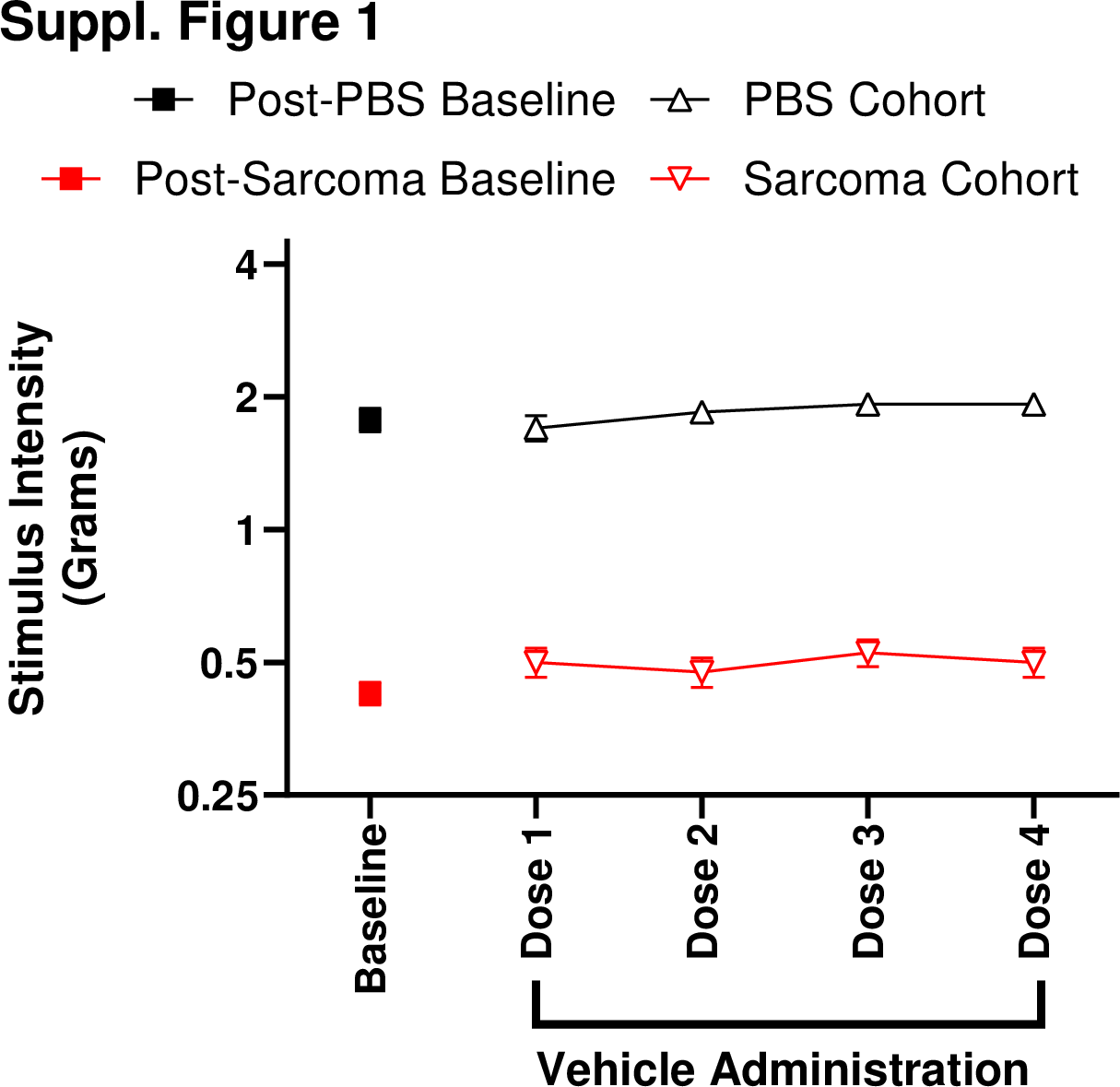

Supplement: S1 Fig — Mechanical allodynia is not impacted by the act of repeated ip injections in repeated administration experiments and any effects on mechanical allodynia are solely an effect of the compound used for treatment. Abscissae: vehicle dose; ordinate: minimum force threshold in grams required to elicit a paw withdrawal response. Data reflect mean ± SEM, n = 8. (TIF) [file pone.0309398.s001.tif]

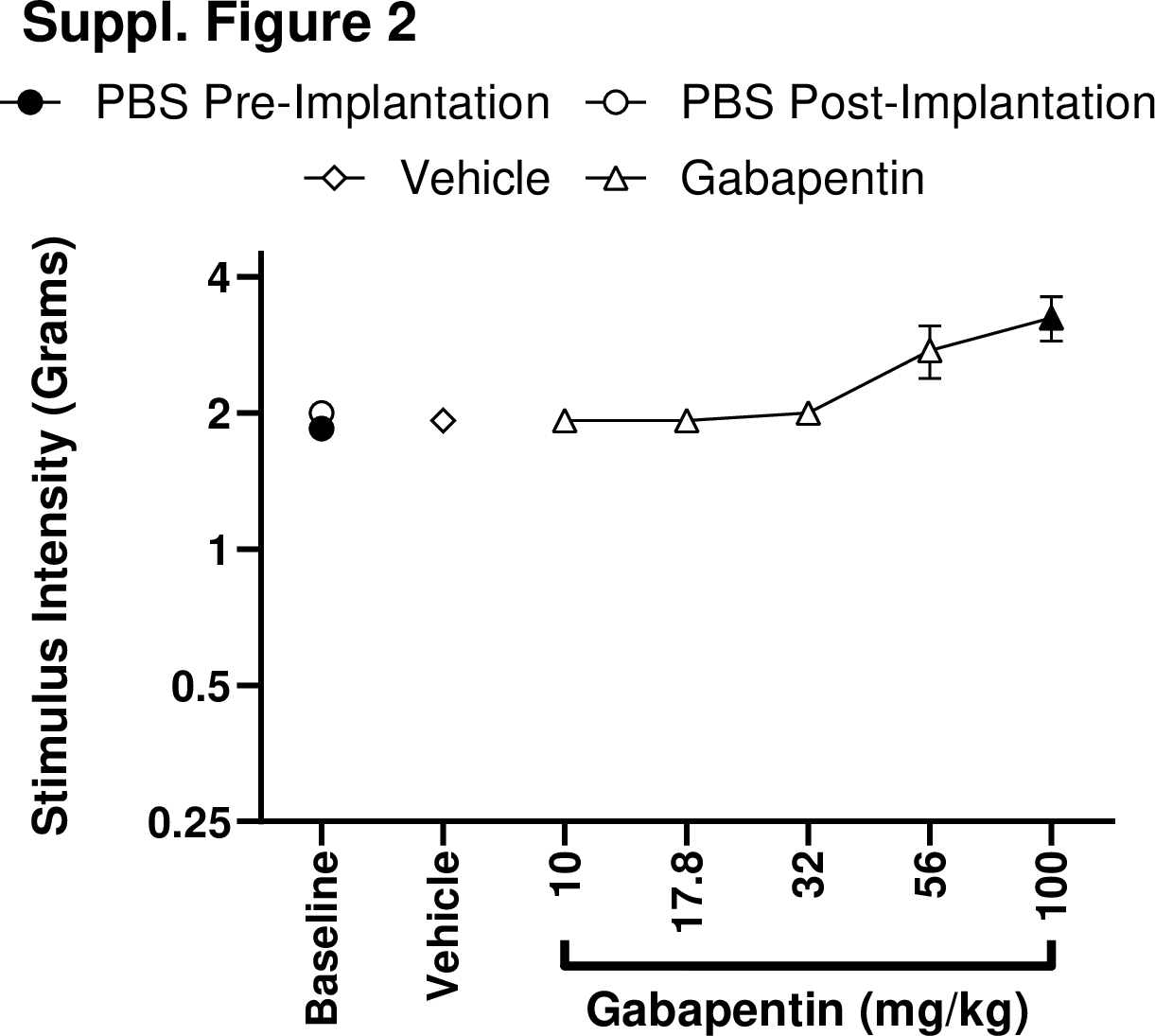

Supplement: S2 Fig — Gabapentin was found to significantly produce analgesic effects at 100 mg/kg Gabapentin via cumulative dosing in the PBS implanted cohort. Abscissae: vehicle dose; ordinate: minimum force threshold in grams required to elicit a paw withdrawal response. Filled data points indicate a significant difference from vehicle treatment (p < 0.05). Data reflect mean ± SEM, n = 8. (TIF) [file pone.0309398.s002.tif]
